# Advanced Material Against Human (Including Covid‐19) and Plant Viruses: Nanoparticles As a Feasible Strategy

**DOI:** 10.1002/gch2.202000049

**Published:** 2020-12-28

**Authors:** Gonzalo R. Tortella, Olga Rubilar, María Cristina Diez, Jorge Padrão, Andrea Zille, Joana C. Pieretti, Amedea B. Seabra

**Affiliations:** ^1^ Centro de Excelencia en Investigación Biotecnológica Aplicada al Medio Ambiente CIBAMA‐BIOREN Universidad de La Frontera Temuco 4811230 Chile; ^2^ Chemical Engineering Department Universidad de La Frontera Temuco 4811230 Chile; ^3^ Centre for Textile Science and Technology (2C2T) University of Minho Guimarães 4800‐058 Portugal; ^4^ Center for Natural and Human Sciences Universidade Federal d ABC (UFABC) Santo André 09210‐580 Brazil

**Keywords:** COVID‐19, human viruses, nanobiotechnology, nanoparticles, plant viruses

## Abstract

The SARS‐CoV‐2 virus outbreak revealed that these *nano‐pathogens* have the ability to rapidly change lives. Undoubtedly, SARS‐CoV‐2 as well as other viruses can cause important global impacts, affecting public health, as well as, socioeconomic development. But viruses are not only a public health concern, they are also a problem in agriculture. The current treatments are often ineffective, are prone to develop resistance, or cause considerable adverse side effects. The use of nanotechnology has played an important role to combat viral diseases. In this review three main aspects are in focus: first, the potential use of nanoparticles as carriers for drug delivery. Second, its use for treatments of some human viral diseases, and third, its application as antivirals in plants. With these three themes, the aim is to give to readers an overview of the progress in this promising area of biotechnology during the 2017–2020 period, and to provide a glance at how tangible is the effectiveness of nanotechnology against viruses. Future prospects are also discussed. It is hoped that this review can be a contribution to general knowledge for both specialized and non‐specialized readers, allowing a better knowledge of this interesting topic.

## Graphical abstract

Viruses can cause important global impacts, affecting public health, and agriculture. The use of nanotechnology for viral disease treatments has played an important role. This technnology has been used in drug delivery, treatments of some human viral diseases, and as antivirals in plants. An overview of the progress during the 2017–2020 period is presented.

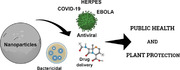

## 1 Introduction

Viral infections have always proved to be a daring challenge to science and public health, due to their plasticity and their “hostile takeover” of the metabolic machinery of the host cells. In fact, the virus dependence on the cell host metabolic machinery for their propagation greatly limits drug efficacy without non‐negligible cytotoxicity. Then, as well as other pathogenic microorganisms, damage caused by the viruses or “*nano‐pathogens*” present a great concern for human and agricultural health worldwide. From a public health point of view, considerable number of people are affected every year by viral infections. In this regard, respiratory viral infections are the most common diseases identified in hospitalized children.^[^ ^1^ ^]^ Moreover, hepatitis, herpes, or human immunodeficiency infection (HIV) are also an important public concern.^[^ ^2^, ^3^, ^4^ ^]^ In fact, 325 million people worldwide are affected by viral hepatitis leading to 1.34 million deaths annually,^[^ ^5^ ^]^ and HIV is present in more than 36 million people, which means the death of 1 million people every year.^[^ ^6^ ^]^ Another important public concern is the outbreak of new emerging viruses affecting human health. An example is SARS‐CoV detected in 2002, and SARS‐CoV‐2 (COVID‐19) which was first detected in 2019 in Wuhan, China, and spread worldwide.^[^ ^7^ ^]^ RNA viruses, such as SARS‐CoV or COVID‐19, among others, have a high evolution (through mutation, reassortment, or recombination), allowing them a high adaptability to global changes, being able to generate local outbreaks up to pandemic worldwide.^[^ ^8^ ^]^ In this regard, a clear example is H1N1 and H5N1 influenza A virus, which are defined by Jaijvan et al.^[^ ^8^ ^]^ as emergence and reemergence viruses. An important thing about this problem is regarding the atmospheric conditions favoring virus diffusion. In this sense, Coccia^[^ ^9^ ^]^ established a clear high relation between the accelerated and vast diffusion of COVID‐19 in North Italy and air pollution (elevated PM_10_ or ozone) and also low wind.

Other important concerns encompass are the emergence of resistance to the current antiviral therapies or cytotoxicity, short plasma half‐life, low solubility, among others.^[^ ^10^, ^11^ ^]^ Therefore, alternatives to traditional drug therapies are necessary for the treatment of viral infections to overcome these barriers.

On the other hand, from an environmental health point of view, agricultural crops are also affected by viral diseases. Several viruses can infect important crop species resulting in significant economic losses.^[^ ^12^, ^13^ ^]^ Interestingly, more than two viruses can attack one crop at the same time.^[^ ^14^ ^]^ An example is *Geminiviridae* family,^[^ ^15^ ^]^ representing 360 species of plant viruses, and responsible for several diseases on economically important crops.^[^ ^12^, ^14^ ^]^ The main problem in field is that viruses reveal themselves normally via symptoms. The control is restricted to the vectors (insecticides), because the antivirals are scarce, and on many occasions ineffective.^[^ ^14^, ^16^ ^]^ Moreover, their inadequate use can represent a concerning level of environmental pollution,^[^ ^17^ ^]^ given that many chemical compounds used for the treatment of diseases in both public health and agricultural production can represent an environmental and health risk due to their active ingredient, at is shown by SanJuan‐Reyes et al.,^[^ ^18^ ^]^ where clearly are indicated as persistent or toxic compounds commonly used such as quaternary ammonium chloride, sodium hypochlorite, or orthophenylphenol (OPP).

Thermotherapy, crop rotation, or genetically resistant crops are also applied for virus control.^[^ ^19^, ^20^ ^]^ However, novel viral disease management in crops system that allows a more efficient control in field is warranted.

In relation to the aforementioned, the question arises why is it necessary the research of new source of technological solutions? What drives us to seek solutions for the treatment of viral diseases in humans and in crops? The first, is that unsolved problems that cause human and economic losses are a constant concern. However, a relation between the development of technological trajectories, role to relevant problems as well as their solution to explain the emergence of path‐breaking innovations and competitive advantage of firms, is shown.^[^ ^21^ ^]^ About this, the nanotechnologies have been affecting biomedicine and agriculture with groundbreaking applications virology. In fact, several nanotechnological applications shown are effective strategies in diagnostics and treatment (delivering antiviral drugs) of these pathologies^[^ ^22^, ^23^ ^]^ as shown in this manuscript. Moreover, nanotechnology has been supported by continuous growth in several areas such as genetics, genomics, proteomics among others.^[^ ^22^ ^]^ An example of this is the recent advances in nanoparticle‐based lateral flow immunoassays, which are used as easy, simple, rapid, and cost‐effective tools, to be applied in pathogen detection in agriculture and medicine.^[^ ^24^ ^]^

**Figure 1:**
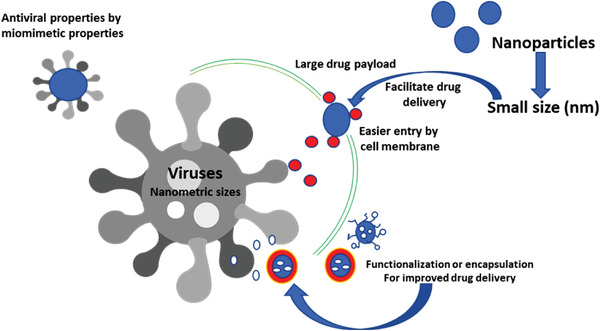
Main proposal mechanisms by which nanoparticles can exert their antiviral properties.^[^ ^23^ ^]^

Due to their size, exceptional properties and practical applications in many areas, nanoparticles and nanomaterials are a fascinating alternative to overcome the challenges caused by viral infections in both medicine and agriculture applications. But, why are nanoparticles so attractive to be used as an antiviral tool? **Figure** **1** describes the main mechanisms by which nanoparticles can exert their antiviral properties.^[^ ^23^ ^]^ These outstanding properties have allowed developing of many new strategies in this “nano‐war” against human and plant viruses. Undoubtedly, human health is a priority from every point of view. However, plants, crops in particular, are also vital to human health. In addition, viral diseases are difficult to treat, and lead to economic losses, due to poor‐quality and lower yield production,^[^ ^25^ ^]^ mainly affecting countries that depend on their agricultural production.

Therefore, this review provides an overview including some of the most recent reports (2017–2020) in which biotechnology has played an important role for public health and agriculture, for the progress in the treatment of viral diseases. We present the recent progress in the design and application of nanoparticles as drug delivery carrier, and as a potential treatment for human and plant viral infections not only using texts but also with Figures and Tables. This review is aimed at both specialized and non‐specialized readers interested in this spellbinding topic. When adequate, the reader will be referred to others with very interesting and specific reviews, for a more specialized lecture.

## 2 Nanoparticles as Nanocarriers for Antiviral Drugs

**Figure 2:**
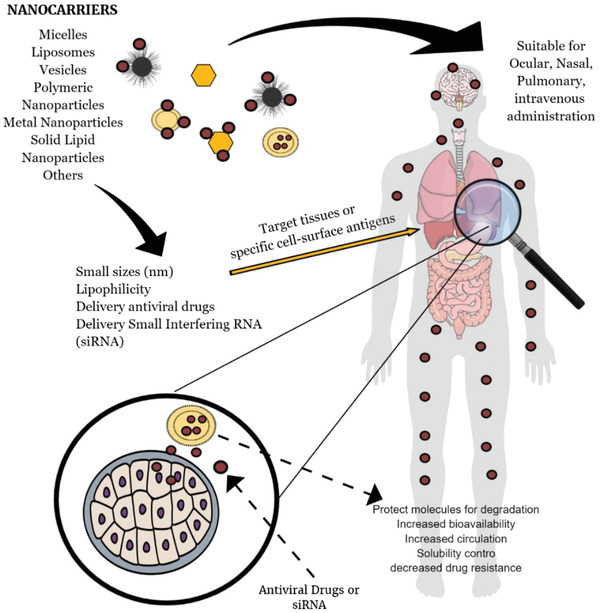
The main advantages of nanoparticles as carrier antiviral drug delivery.

Low bioavailability represents a major limitation for several antiviral drugs, which may occur due to their active excretion or by exhibiting hydrophobicity and/or lipophobicity. Limited bioavailability usually depicts: reduced plasma half‐life, not achievable half‐maximal inhibitory concentration (IC50), and a lower concentration threshold for drug crystallization, which may cause a grievous acute renal failure. Acyclovir, zidovudine, efavirenz, cidofovir, tenofovir, lopinavir, among other antiviral drugs display low bioavailability. Moreover, antiviral drugs must be able to permeate through main body barriers to reach important virus reservoirs. This is particularly important for acquired immunodeficiency syndrome AIDS treatment since major human immunodeficiency viruses (HIV) reservoirs are present in the brain, bone marrow, and adipose tissue.^[^ ^26^ ^]^ Therefore, the development of nanocarriers for antiviral drugs has already proved to be efficient in circumventing or minimizing the most relevant limitations of antiviral drugs, particularly through their tailorable size. The main characteristics of each nanoparticle will influence the mechanisms involved in drug delivery.^[^ ^27^ ^]^ However, their use as nanocarrier represents some advantages that are shown in **Figure** **2**. Nanocarriers with an average size between 200 and 30 nm have a higher potential to avoid immediate renal extraction and consequently to possess a higher plasma half‐life. In addition, nanocarriers comprising a mean size ranging from 50 to 250 nm favorable to blood–brain barrier permeation.^[^ ^28^ ^]^ One of the most commonly and recently used nano solutions are the solid lipid nanoparticles (SLNPs) and poly (lactic‐*co*‐glycolic acid) nanoparticles (PLGANPs). SLNPs are able to carry a relevant quantity of lipophilic antiviral drugs in their lipid core, which is stabilized by a surfactant. Antiviral drug entrapment efficiency (EE) and loading capacity (LC) of SLNPs vary due to lipid and surfactant (and co‐surfactant when present) composition, nevertheless the smaller size of SLNPs is indicated as a crucial to obtain higher EE and LC, due to the SLNPs higher surface area.^[^ ^29^, ^30^, ^31^, ^32^, ^33^, ^34^, ^35^ ^]^ One of the most interesting PLGA‐NPs property is their highly tailorable nature, with a considerable described methodology produce PLGANPs capable to carry lipophilic or hydrophilic molecules of different sizes and structural motifs, and assures its protection. In addition, PLGA‐NPs surfaces are readily modifiable.^[^ ^36^, ^37^, ^38^ ^]^ For instance, the functionalization of PLGANPs with a maleimide‐polyethylene glycol (PEG) improved plasma half‐life by providing stealthiness towards reticuloendothelial system.^[^ ^37^ ^]^ Valine functionalization of PLGA‐PEG nanoparticles allowed its recognition by intestinal transporters improving the PLGA‐PEG‐valine nanoparticles residence time and enhanced the permeability of carried acyclovir.^[^ ^38^ ^]^ PLGANPs have also been used as a carrier for combined antiviral drugs, encompassing up to three different antiviral drugs, and a formulation showed viability to be applied subcutaneously, which may adapt to a wearable injector device, which would help prevent patient nonadherence.^[^ ^39^, ^40^, ^41^ ^]^

## 3 Human Viral Diseases and Nanoparticles

In the past 30 years, many antiviral agents have been formulated as a possible therapy.^[^ ^42^, ^43^ ^]^ However, in some cases, these drugs are ineffective or they can cause critical side‐effects.^[^ ^44^, ^45^ ^]^ Drug resistance has also been reported.^[^ ^10^, ^11^ ^]^ Therefore, new effective therapies are required. In the last ten years, nanoparticles have emerged as an important tool to combat diseases.^[^ ^46^ ^]^ Due to their interesting and unmatched properties, nanoparticles can be used in different applications ranged from biocides to nanocarriers for antiviral drugs. During the last ten years, many studies on viral diseases involving nanoparticles have been published, as reviewed by Sing et al.^[^ ^23^ ^]^ and Khezerlou et al.^[^ ^47^ ^]^ Indeed, antiviral nano formulations (under evaluation or clinically approved) are depicted by Singh et al.^[^ ^23^ ^]^ The next sections present different antiviral applications of nanoparticles.

### 3.1 Nanoparticles for Viral Respiratory Diseases

Viral respiratory pathologies have been a severe problem for public health, causing many deaths worldwide.^[^ ^48^, ^49^ ^]^ Indeed, pandemic influenza in 1918–1919 period caused more than 25 million deaths,^[^ ^50^ ^]^ and currently, COVID‐19 pandemic led to more than 3 million infected people worldwide, and 216 000 deaths until the end of April 2020.^[^ ^51^ ^]^ Nanobiotechnology offers potential solutions for viral diseases, and some promissory results are summarized in **Table** **1**.

**Table 1:** Some recent examples of nanoparticles evaluated against respiratory viruses

Virus or viral disease	Nanoparticle	Relevant Finding	Reference
Influenza Virus	AgNPs/ virus‐inactivated flu vaccine	Increase of immunoglobulin A and antibodies titers without toxicity and reduction of viral load in mice	^[^ ^52^ ^]^
Respiratory syncytial virus (RSV)	AgNPs	Increase in cherokine (CXCL1), granulocyte colony‐stimulating and granulocyte‐macrophage colony stimulating factors and decrease in pro‐inflammatory cytokines and chemokines	^[^ ^53^ ^]^
H1N1 influence	Fe_3_O_4_	50% in virus reduction after 72 h, and decrease in viral RNA transcripts (measured as antiviral activity).	^[^ ^54^ ^]^
	phosphate nanoparticles	As adjuvant in vaccine caused virus neutralization, high lgG antibodies, and higher inhibition of hemagglutination	^[^ ^55^ ^]^
	PEG‐coated ZnNp	Viral inhibition rate > 90%, significant reduction in TCID50 (Median Tissue Culture Infectious Dose)	^[^ ^56^ ^]^
H1N1, H3N2 and H5N1 virus	AuNp/M2e peptide	Intranasal delivery in mice increased lung C cell activation and lgG1 and lgG2a subtypes. 100% protection for H1N1 and H5N1 and 92% for H3N2. Generated antibodies were binding to M2 expresses in infected cells.	^[^ ^57^ ^]^
H1N1 and H3N2 virus	Two mixed nanoparticles as vaccine: a) protein matrix (M2c)/nucleoprotein antigens and b) M2c and papaya mosaic virus	Multimerization of nucleoprotein antigens on nanoparticles caused strong protection and significant immune response against the two viruses	^[^ ^58^ ^]^
H5N1, H1N1, H5N2, H7N3 and H9N2	Double‐layered protein chitosan nanoparticle or cholera toxin subunit A1, matrix protein‐2 (sM2), fusion peptide of hemagglutinin (HA2), and poly‐γ‐glutamic acid‐chitosan nanoparticles	High immunity against all viruses.	^[^ ^59^, ^60^ ^]^

Silver nanoparticles (AgNPs) have demonstrated antiviral activity.^[^ ^53^, ^61^, ^62^, ^63^ ^]^ SARS‐CoV‐2 virus is the most recent pandemic worldwide. Social mitigation strategies and non‐specific drug therapies have been implemented.^[^ ^64^, ^65^, ^66^, ^67^, ^68^, ^69^ ^]^ Given that this is an emerging virus, studies involving nanoparticles are still scarce. However, Zachar et al.^[^ ^70^ ^]^ reported that inhalation delivery of AgNPs favored the response time of immune system. Potential action mechanisms of chloroquine on COVID‐19 has been possible using synthetic nanoparticles.^[^ ^71^ ^]^ In addition, Aydemir and Ulusu^[^ ^72^ ^]^ reported that angiotensin‐converting enzyme 2 coated nanoparticles are an effective tool to be used in protective element to avoid the spread of the COVID‐19.

Finally, given that no specific vaccines or drugs have been developed yet for the COVID‐19 treatment, it is important to mention that nanotechnology has nevertheless offered a series of tools that can be used as biosensors for its detection. In this sense, gold,^[^ ^73^ ^]^ lantanide‐doped polystyrene,^[^ ^74^ ^]^ or magnetic nanoparticles^[^ ^75^ ^]^ and graphene^[^ ^76^ ^]^ are presented as interesting nanostructures to be used as biosensor for COVID‐19 detection as reviewed by Srivastava et al.^[^ ^77^ ^]^

### 3.2 Nanoparticles for Viral Skin Diseases

AgNPs have demonstrated a high capacity for controlling herpes simplex virus types 1 and 2 (HSV‐1 and HSV‐2).^[^ ^78^ ^]^ Results in mice showed that AgNPs modified with tannic acid, applied intravaginally, presented lower virus titers in the tissues, and a significant increase in the immune response.^[^ ^79^ ^]^ Effective control of γ‐herpesvirus‐related cancer cell with AgNPs demonstrated a higher cytotoxicity against infected cells with Kaposi's sarcoma‐associated herpesvirus (KSHV or HHV‐8) or Epstein–Barr Virus (EBV or HHV‐4) through reactive oxygen species (ROS) generation and autophagy.^[^ ^61^ ^]^ Biogenic AgNPs have also demonstrated antiviral effects against HSV virus, decreasing in more than 40% the cytopathic effect of HSV‐1 and HSV‐2.^[^ ^80^ ^]^

Sulfonated magnetic nanoparticles functionalized with graphene oxide allowed an efficient capture of HSV‐1, due to the higher photothermal antiviral activity (≈99.99%).^[^ ^81^ ^]^ Studies in Vero‐cells demonstrated a high effectivity of gold nanoparticles (AuNPs) against HSV‐1 and HSV‐2, preventing viral attachment and cell penetration.^[^ ^82^ ^]^ AuNPs have the capacity to cross de blood–brain barrier in mice model, without cerebral damage, and inhibiting HSV‐1 infection.^[^ ^83^ ^]^ Interestingly, the authors reported that AuNPs also reduced HSV‐1 induced β‐secretase activity, which is related to Alzheimer's disease.^[^ ^84^ ^]^ Copper nanoparticles (CuNPs) at 100 µg mL^−1^ significantly decreased virus titers (HSV‐1) with an inhibition rate of 83.3%.^[^ ^85^ ^]^ In an interesting work, Lee et al.^[^ ^86^ ^]^ demonstrated that nanoparticles modified with glycosaminoglycan attached to mesoporous silica nanoparticles, and loaded acyclovir, were efficient against HVS‐1 and HVS‐2, given that simultaneous target such as inhibition of viral entry and inhibition of DNA replication occurred. Natural nanoparticles have also been demonstrated to be efficient in the control of HSV‐1. Shen et al.^[^ ^87^ ^]^ reported that nano powder of *Rheum tanguticum* roots could interfere with the entire phase of viral replication of HSV‐1 virus, inhibiting the infection through several mechanisms.

### 3.3 Nanoparticles for HIV‐1 infection

Thousands of works have been reported on the use of nanoparticles as an emerging antiviral therapeutic agent in the war against HIV‐1 (such as drug delivery carriers, vaccines coadjutant, among others). Therefore, we will give to the readers some examples in the recent progress of the uses of nanoparticles as antiviral agent or as carrier for drug delivery. For more detailed and specialized information, we recommend Gao et al.,^[^ ^88^ ^]^ Rezaeia et al.,^[^ ^89^ ^]^ and Thalhauser et al.^[^ ^90^ ^]^

The mimic of the bind site of HIV‐1 virus in CD4 T lymphocytes has been a strategy against HIV‐1. The use of surface‐modified lactoferrin nanoparticles, conjugated with sodium 2‐mercaptoethanesulfonate, allows to mimic the bind site between HIV‐1 viral protein gp120 and CCR5 (chemokine) and heparin sulfate (anionic polysaccharide) avoiding the infection.^[^ ^91^ ^]^ Moreover, loading nanoparticles with antiretroviral agents allows a controlled release of high effective concentration of the drug in the infected zone. HIV‐1 proteases (HIV‐1 PR) are important and essential enzymes for the life‐cycle of HIV‐1. In this regard, AgNPs efficiently inhibited HIV‐1 PR activity by specific bind to synthetic peptides with similar amino acid sequences to viral polyproteins.^[^ ^92^ ^]^ The use of small interfering RNA (siRNA) has been evaluated to treat diseases as an important strategy to regulate gene expression (RNA interference).^[^ ^93^ ^]^ Chitosan nanoparticles modified with carboxymethyldextran or polyethylenimine were used as carrier delivery of anti‐*tat* siRNA. These nanoparticles in mammalian cell line expressing HIV‐1 Tat (transactivator of transcription) increased the cell viability and anti‐*tat* siRNA delivery efficiency. Moreover, RNA and protein expression of HIV‐1 Tat was significantly reduced.^[^ ^94^ ^]^ In the earliest stages of viral gene expression of HIV‐1, an encoded important protein is Nef, which is critical in HIV‐1 pathogenesis and considered a substantial virulence factor.^[^ ^95^, ^96^ ^]^ Carboxymethyl dextran‐trimethyl chitosan‐coated superparamagnetic iron oxide nanoparticles have shown to be an efficient carrier for delivery anti‐nef siRNA, significantly reducing the expression of HIV‐1 nef in cells.^[^ ^96^ ^]^ Other HIV‐1 inhibitors such as peptide (E2) (peptide derived from GB virus type C)^[^ ^97^ ^]^ have been effectively loaded in polymeric nanoparticles covered with glycol‐chitosan, and with effective mobility in vaginal mucus.^[^ ^98^ ^]^ The envelope (*Env*) trimeric glycoprotein (GP) of HIV‐1 is composed of surface gp120 and transmembrane gp41 subunits organized as trimer of heterodimers, which has several mechanisms to evade the host antibodies.^[^ ^99^ ^]^ A widely studied strategy has been the immobilization of *Env* in a stable surface to be used as a potential vaccine. In this regard, Thalhauser et al.^[^ ^100^ ^]^ reported the effective immobilization of an *Env* variant (BG505 SOSIP.664) on a silica nanoparticle as a potential vaccine platform, and it was demonstrated that *Env* variant showed structural integrity after their attachment. AuNPs have also been satisfactorily used as carrier of antiviral compounds. In this sense, AuNPs decorated with glycans were loaded with HIV‐1 peptides and mannosides‐type oligosaccharides, and evaluated on dendritic cell (specialized antigen‐presenting cells).^[^ ^101^ ^]^ The results showed an increased proliferation of HIV‐specific CD4 and CD8 T cell, and a high secretion of functional cytokine due to functionalized AuNPs. Therefore, these strategies might be a promising approach to enhance vaccines against HIV‐1.^[^ ^101^ ^]^ Nevertheless, further studies are required.

### 3.4 Nanoparticles for Hepatitis Virus Infection

In recent years, several studies involving nanoparticles have reported satisfactory results for the control of HBV‐A and HBV‐B A potential vaccine based in alginate‐coated chitosan nanoparticles (ACNp) loaded with hepatitis B surface antibodies (HBsAg) and anchored with lipopolysaccharide, as adjuvant for oral mucosal immunization, showed that HBsAg was protected by the nanoparticles, and it was detected that a significant production of immunoglobulin A (sIgA) in mucosal, and IgG antibodies in blood circulation.^[^ ^102^ ^]^ Similar results were reported by AbdelAllah et al.,^[^ ^103^ ^]^ where ACNp was evaluated as effective adjuvant for hepatitis A. Nanoparticle application in mice showed production of interferon gamma (INF‐γ), interleukin‐10 (IL‐10), and an increase in seroconversion rate (100%), splenocyte proliferation, and hepatitis A antibodies level. Given that HBV can cause hepatocellular injury, polypeptide penetratin‐based hybrid nanoparticle system carrying IL‐22 gene demonstrated that locally IL‐22 protein (interleukin‐22) expression and activation of STAT3 signaling pathway allowed to repair hepatocyte injury.^[^ ^104^ ^]^ Local expression of IL‐22 activated STAT3/Erk signal, inhibiting ROS accumulation, promoting hepatocyte regeneration without side‐effects in mice were observed. Wang et al.^[^ ^105^ ^]^ evaluated a vaccine in mice, which is based on ferritin nanoparticles and the delivery of the main hepatitis B antigen (pre S1) to specific myeloid cells, which are a critical arm of the immune system. The results demonstrated that high and persistent levels of anti‐preS1 antibodies were induced by the vaccine.

Advances in the treatment of hepatitis C (HCV) by using nanoparticles has also been reported. The subunit E1, an envelope GP of hepatitis C virus, and sE2 (soluble E2) vaccine produced high neutralizing antibodies that can prevent HCV in mice.^[^ ^106^ ^]^ To increase the sE2 immunogenicity, Yan et al.^[^ ^107^ ^]^ evaluated the fusion of sE2 (surface‐displayed), ferritin unit in S2 cells (cell lines) self‐assembled into a nanoparticle. Studies carrying out with mouse immunization revealed that the fusion of sE2‐ferritin into nanoparticle was more efficient than sE2 in the induction of broadly neutralizing antibodies against HCV infection. Recently, He et al.^[^ ^108^ ^]^ developed a potential vaccine strategy for hepatitis C virus (HCV) treatment, redesigning E2 cores on ferritin nanoparticles. The authors reported that in mice a more effective neutralizing antibodies response was obtained with the use of the nanoparticles.

### 3.5 Nanoparticles for Vector‐Borne Diseases

Vector‐borne diseases are an important public health concern, some of them without a specific therapy, and the treatments are focused on symptomatic and supportive care.^[^ ^109^, ^110^ ^]^ **Table** **2** summarizes some relevant findings obtained with nanoparticles for vector‐borne diseases.

**Table 2:** Example of some relevant findings obtained with nanoparticles for vector‐borne diseases

Vector‐borne disease	Nanoparticles	Relevant finding	Reference
Chikungunya virus	AgNPs	Cell viability of infected cells increased 25% and 80%	^[^ ^111^ ^]^
	ZnONp	Cell viability of infected cells increased 90%	^[^ ^112^ ^]^
Zika virus	Upconverting Gd_2_O_3_:Tb^3+^/Er^3+^	Humoral response in mice was enhanced	^[^ ^113^ ^]^
	SiNP functionalized with zwitterion and biological active group	Prevention of nonspecific protein adhesion and shielding effects against virus	^[^ ^114^ ^]^
Ebola virus	Nickel nitrilotriacetic acid‐functionalized lipid nanoparticles combined with rGP antigen	Virus neutralization by stimulated germinal center B cells and polyfunctional T cell	^[^ ^115^ ^]^
?	EBOV GP nanoparticle vaccine with the saponin‐based adjuvant Matrix‐M (Novavax, Inc.)	Anti‐EBOV GP immunoglobulin G titers were produced quickly in healthy adults. Assays with wild‐type Zaire EBOV or pseudovirion were ninefold higher of Serum EBOV‐neutralizing and binding antibodies	^[^ ^116^ ^]^
Dengue virus	AuNPs/EDIII GP dengue virus	High level of serotype‐specific neutralization of dengue virus in BALB/c mice.	^[^ ^117^ ^]^
	PLGA nanoparticles/ tetravalent dengue virus protein subunit (rE)	High induction of neutralizing antibody, and a most balanced serotype specific antibody response to each dengue virus	^[^ ^118^ ^]^
	Curcumin‐based nano emulsion	Improve solubility, cell uptake efficacy of curcumin, and dengue virus inhibition.	^[^ ^119^ ^]^

## 4 Effects of Nanoparticles on Viral Infections in Plants

Many viruses affect a wide range of crops and lead to huge losses in terms of economy and food supply. Recently, top 10 scientifically and economically significant plant viruses were nominated considering 250 votes from the international community.^[^ ^120^ ^]^ The top 10 were ranked as follows: 1) Tobacco mosaic virus (TMV), 2) Tomato spotted wilt virus, 3) Tomato yellow leaf curl virus, 4) Cucumber mosaic virus, 5) Potato virus Y, 6) Cauliflower mosaic virus, 7) African cassava mosaic virus, 8) Plum pox virus, 9) Bromemosaic virus, and 10) Potato virus X.^[^ ^120^ ^]^ As these viral infections may represent 15% loss of food supply, new strategies are inherent to prevent and treat, at least, the main affected crops.^[^ ^121^ ^]^ The use of nanoparticles in agriculture has been increasing during the last few years. It is already well‐known that at ideal concentrations, nanoparticles of different compositions are able to improve plants’ physiology, increasing plant growth, development, and managing stress and bacterial, fungal, and viral infections.^[^ ^122^, ^123^, ^124^ ^]^

**Figure 3:**
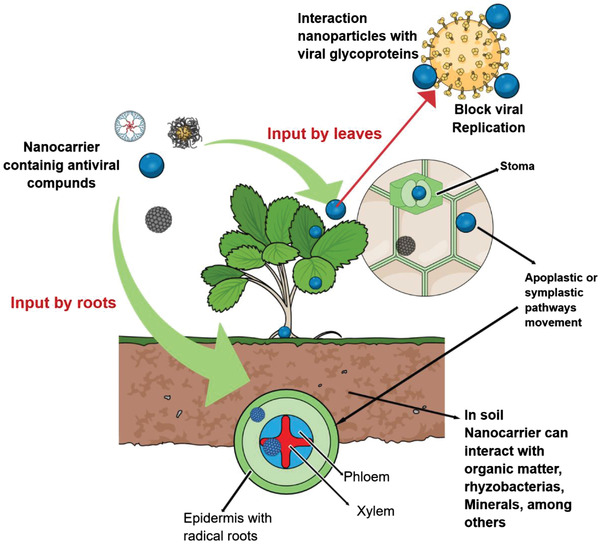
Schematic representation of nanoparticles uptake in plants and their interaction with viral hosts.

Nanoparticles can be applied through foliar or root application, being able to penetrate cell wall and cell membrane, translocating to arterial parts and leaves.^[^ ^125^ ^]^ The uptake is dependent on various factors such as soil, route exposure, and the intrinsic properties of nanoparticles (size, morphology, and surface features).^[^ ^125^ ^]^ When plants are infected by virus, there is the accumulation of viral proteins in host cells, enabling various roles of replication, movement, and suppression of host defense.^[^ ^126^ ^]^ Nanoparticles are presented as an opportunity of combating viral infections in plants, owing to the ability of strong and direct interactions of the nanomaterials with viral surface GPs, enabling their access to the cells, interaction with the genoma and, blocking viral replication (**Figure** **3**).

### 4.1 Current Progress in the Administration of Nanoparticles against Plant Viral Infection

Research englobing nanoparticles and agriculture has exponentially grown in the last decade, evidenced by 25‐fold more articles published in this area in 2019 when compared to 2011, as shown by Web of Science database. Even though, in general, papers englobing the treatment of viral infections in crops using nanoparticles have not accompanied the general tendency of agricultural applications.^[^ ^127^ ^]^ The application of nanoparticles against virus infection in plants is still unexplored and there is plenty of room to be studied.^[^ ^128^ ^]^ Among the nanostructures, AgNPs have been the most studied against viral infections in different crops.

**Table** **3** summarizes the recent advances in the treatment of diverse plant viral infections with nanoparticles. AgNPs had already been reported as promising antiviral agent in general applications.^[^ ^129^ ^]^ The antiviral potential of AgNPs has been demonstrated against Sunhemp rosette virus‐infected guar beans (*Cymopsis tetragonaloba*).^[^ ^130^ ^]^ AgNPs (15 nm) synthesized with a spore crystal mixture of *Bacillus thurigiensis* were spray applied in contaminated leaves of guar beans in the concentration of 50 mg L−^1^. After 3–4 days of inoculation of Sunhemp rosette virus, untreated plants demonstrated 100–150 lesions, while plants treated with AgNPs had no virus lesions, indicating that AgNPs led to complete suppression of the viral infection.^[^ ^130^ ^]^ Similarly, AgNPs synthesized by *Bacillus pumilus*, *Bacillus persicus*, and *Bacillus licheniformis* led to 77–92 nm particles.^[^ ^131^ ^]^ Bean yellow mosaic virus contaminated fava beans were treated with 0.1 µg µL^−1^ of AgNPs using two different strategies of application: i) administration of the nanoparticles 72 h prior to inoculation and ii) administration of the nanoparticles 24 h post‐inoculation. Interestingly, the pre‐treatment did not positively or negatively affect the viral infection, whereas the post‐treatment prevented all symptoms caused by the Bean yellow mosaic virus.^[^ ^131^ ^]^

**Table 3:** Summary of relevant publications based on the use of nanoparticles against different viral infection in crops

Virus	Crop	NPs composition	NPs size	Biological effect	Ref
Tobacco mosaic virus	*Nicotiana benthamiana*	ZnO NPs and SiO_2_ NPs	55/62 nm	Inactivation of TMV and inhibition of viral	^[^ ^134^ ^]^
Tomato mosaic virus/ Potato virus Y	*Lycopersecon esculantum*	AgNPs	20–100 nm	NPs binded to virus coat protein and decreased virus concentration	^[^ ^132^ ^]^
Bean yellow mosaic virus	*Vicia faba*	AgNPs	77–92 nm	Decreased virus concentration and percentage of infection	^[^ ^131^ ^]^
Turnip mosaic virus	*N. benthamiana*	Fe_2_O_4_, TiO_2_, carbon nanotubes, fullerene	40–100, 20, 30 and 50 nm	NPs inhibited vírus proliferation and suppressed viral infection	^[^ ^127^ ^]^
Sunhemp rosette virus	*Cymopsis tetragonaloba*	AgNPs	15 nm	Complete suppression of viral infection	^[^ ^130^ ^]^
Tomato spotted wilt virus	*Chenopodium amaranticolor*	AgNPs	12 nm	Inhibitory effects were higher when NPs were sprayed after virus inoculation	^[^ ^133^ ^]^
Potato virus Y	*Solanum tuberosum L*.	Curcumin‐milk proteins NPs	235–335 nm	Concentration‐dependent virus inhibition	^[^ ^135^ ^]^

Commercial or conventionally synthesized AgNPs also demonstrated significative antiviral properties against *Tomato mosaic virus, Tomato spotted wilt virus*, and *Potato virus Y*.^[^ ^132^, ^133^ ^]^ Divergent results were verified for tomato plants contaminated with both Tomato mosaic virus and Potato virus Y when compared to the ones contaminated with *Bean* yellow mosaic virus.^[^ ^131^, ^132^ ^]^ AgNPs at 50 ppm were spray applied 7 days before virus inoculation, and the results showed that the disease severity was significantly decreased for both viruses.^[^ ^132^ ^]^ On the opposite, the treatment using AgNPs on potato plant infected with *Tomato spotted wilt virus* demonstrated that the best treatment condition was 24 h post virus inoculation.^[^ ^132^, ^133^ ^]^

Other studies have been based on the use of metal oxide and carbon‐based nanomaterials.^[^ ^127^, ^134^ ^]^ In vitro evaluation of biological effect of ZnO NPs (20 nm) and SiO_2_ NPs (18 nm) against Tobacco mosaic virus were performed and compared to the effect of conventional agent letinan (LNT), currently used as antiviral agent.^[^ ^134^ ^]^ Both ZnO NPs and SiO_2_ NPs led to more pronounced toxicity than the LNT. In vivo experiments confirmed that the nanoparticles remarkably inhibited virus replication, not only acting on plant defense but also increasing dry and fresh weights.^[^ ^134^ ^]^ Hao and coworkers evaluated the potential of different nanomaterials against viral infection in *Nicotiana benthamiana*.^[^ ^127^ ^]^ The plants were pre‐treated with Fe_2_O_3_ or TiO_2_, and with multi‐walled carbon nanotubes (MWCNT) or fullerene. Two different concentrations (50 and 200 mg L^−1^) of each nanoparticle were foliar sprayed for 21 days prior to virus inoculation. All nanoparticles presented relative suppression of the viral infection, although results were more pronounced for MWCNT at 50 mg L^−1^.^[^ ^127^ ^]^ Taha et al. demonstrated the potential of milk‐proteins nanoparticles loaded with curcumin against Potato virus Y.^[^ ^135^ ^]^ The nanoparticles were sprayed at different concentrations after 7 and 14 days of infection. Results indicated a dose‐dependent inhibition, reaching 100% at the highest evaluated concentration (1500 mg mL^−1^).^[^ ^135^ ^]^

This literature review reveals two major points: i) there is strong evidence of the positive effects that nanomaterials present in the treatment of virus infections in plants, and ii) the results are divergent and not standardized. From the highlighted papers, all were performed with a different type of virus and a different crop. Furthermore, the types of nanoparticles employed were not only variated but also used in different concentrations and applied for different periods of time, pre‐ and post‐virus inoculation. Thus, there is a strong need for standardization in the evaluation of the antivirus effects of nanoparticles in plants. The antivirus effects of nanoparticles in plants depend on: 1) virus type, 2) crop, 3) nanoparticle composition, and 4) treatment protocol. Thus, further work should be developed to better understand the mechanisms and potential of different nanoparticles against a variety of viral infections in important crops, highlighting the ideal concentration ranges and the frequency/moment of application.

### 4.2 Unexplored Nanoparticles with Promising Potential against Crop Viral Infection

Amongst different nanoparticles for agricultural applications, some examples should be highlighted as promisor candidates for future advances in viral infections management. A largely studied example is the use of chitosan nanoparticles (CS NPs). Recently, CS NPs have shown important effects in various fields of agriculture, such as pest management, fertilization, and improvement of plants in drought conditions.^[^ ^136^, ^137^, ^138^ ^]^ Generally, the action of CS NPs in each application is intrinsically related to the content carried by those particles. The management of pests was provided for CS NPs loaded with agrochemicals or, alternatively, essential oils.^[^ ^138^, ^139^ ^]^ Montmorillonite clay and potassium nitrite loaded CS NPs were used for fertilization, while drought tolerance was improved by nitric oxide (NO) loaded nanoparticles.^[^ ^136^, ^137^ ^]^ Similar strategies are also applied to other polymeric nanoparticles that act as capsules for controlled release, such as acid‐based PLGA‐NPs which were used as carriers of antiviral ribavirin.^[^ ^140^ ^]^ Results indicated that small nanoparticles (>100 nm) were successfully synthesized and that a sustained release of ribavirin, confirming the potential for future applications in the management of virus in crops.^[^ ^140^ ^]^ Thus, the administration of polymeric nanoparticles represents a promising strategy for future studies regarding their applications in the control of viral infections in crops, through a controlled and effective release of antiviral agents.

Metal and metal‐oxide nanoparticles have also been vastly studied in the field of agriculture. Even representing the most studied nanoparticles against viral infections, there is still plenty of room to be explored in this regard. Species such as copper nanoparticles, cerium oxide, and zinc oxide nanoparticles have already demonstrated promising applications in agriculture, however their effectiveness against viral infections, either by directly interacting with the virus or improving plants’ defense, have not been evaluated.^[^ ^137^, ^141^, ^142^ ^]^ Different synthetic routes impact the size and surface properties of each nanoparticle, reflecting nanoparticle adhesion, uptake pathways, and translocation in plants.^[^ ^143^ ^]^ Therefore, not only a larger variety of nanoparticles might be explored, but also the best parameters for each nanoparticle should be evidenced for specific application, considering both crop and virus types.

## 5 NO‐Releasing Nanomaterials as a Promising Weapon against Viral Infection in Plants

The gaseous free radical NO is found in plants and plays important functions in plant defense.^[^ ^144^ ^]^ NO is produced in plants by an enzymatic pathway through the action of a NO synthase (NOS)‐like enzyme and nitrite reductase.^[^ ^145^ ^]^ As small and lipophilic molecule, NO easily diffuses across cell membranes acting as a biological messenger during various aspects of plant development and growth, such as senescence, photomorphogenesis, root growth leaf expansion, floral transition, stomatal closure, vegetative growth, cytokinin signaling, and programmed cell death.^[^ ^145^, ^146^ ^]^ NO is involved in the plant acclimation to different abiotic and biotic stresses including defense against pathogen attack.^[^ ^147^ ^]^ Plant hormones such as salicylic acid, abscisic acid, auxin, jasmonic acid, cytokinin, and brassinosteroids enhance NO production during plant defense against microbial pathogens.^[^ ^145^, ^146^ ^]^ As a signaling molecule, NO mediates brassinosteroid resistance to Cucumber mosaic virus in *Arabidopsis thaliana* ^[^ ^148^ ^]^ and brassinosteroid cross talks with NO triggering the susceptibility of maize to chlorotic mottle virus infection.^[^ ^147^ ^]^ NO and salicylic acid were reported to have a synergist effect against tomato mottle mosaic virus.^[^ ^149^ ^]^ It has been reported that nitrate reductase (an enzyme that reduces nitrite to NO) alleviated cucumber mosaic virus in *A*. *thaliana*, in which synthesized NO regulated salicylic acid to mediate defense response.^[^ ^150^ ^]^

Exogenous administration of NO donors can trigger the expression of genes related to plant defense and thus increasing the generation of ROS inducing cell death. NO has been reported to be involved in the systemic acquired resistance, conferring a broad‐spectrum disease resistance to infections.^[^ ^151^ ^]^ Moreover, NO can S‐nitrosate cysteine thiol moieties of important biomolecules involved in the plant defense signaling pathway.^[^ ^152^ ^]^

Recently, Lu et al reported the pretreatment of rice with the NO donor, sodium nitroprusside (100 µM), followed by infection with rice stripe virus.^[^ ^153^ ^]^ The authors demonstrated that NO and melatonin play a key role in the virus‐plant interaction in a synergistic manner. The administration of NO inhibitors or NO scavenger enhanced the incidence of rice stripe virus, confirming plant defense role of NO.^[^ ^143^ ^]^

Sodium nitroprusside associated with salicylic acid promoted plant (*Arachis hypogaea* L.) resistance against *Peanut mottle* virus infection. The plant resistance was accomplished by an increase in the photosynthetic pigments, an increase in the activity of antioxidant enzymes, a superior content of oils at the harvest time, and an increase in the levels of fatty acids, compared with untreated plants.^[^ ^154^ ^]^

Therefore, NO plays a key role in plant defense against pathogens. Although the administration of NO donors has been extensively applied in biomedical applications, more recently in combination with nanomaterials,^[^ ^155^ ^]^ this strategy is poorly explored in plant defense under biotic stress. NO donors and NO‐releasing nanomaterials have been applied in the mitigation of plants under abiotic stresses, such as drought,^[^ ^124^ ^]^ salinity,^[^ ^156^ ^]^ UV‐light exposure.^[^ ^157^ ^]^ However, the administration of NO‐releasing nanomaterials has not been extensively explored in plants under biotic stress. Due to the versatility and importance of NO in plant defense, further investigations should be explored in this promising topic.

## 6 Conclusion and Perspectives

The aim of this review is to provide information to the readers of the most current advances in the use of nanobiotechnology in the war against viruses, that is, application of nanoparticles for the control of viral infections. We have addressed the nanoparticles capacities as carrier of antiviral drugs, and the applications of these for human and plant viral pathologies. Recent progress has been demonstrated that nanoparticles allowed to overcome many challenges imposed through the use of conventional antiviral therapies.^[^ ^158^ ^]^ Nanoparticles allowed to overcome problems such as the cross of the blood barrier in the brain, and through their functionalization, to enhance the affinity of nanoparticle to the antiviral drugs, allowing a sustained and localized drug delivery. Depending on the chemical nature of the nanoparticle, the antiviral drug can be encapsulated in the nanoparticles or added to the nanoparticle surface. As stated in this review, the toxicity of antviral drugs can be decreased upon their loading in a nanoparticle (resulting in fewer side effects).

There are plenty of different nanoparticles that can be explored as antiviral carriers in mammals and in plants, ranging from biodegradable polymeric nanomaterials to rare earth upconverting nanoparticles. Depending on the nanoparticle feature, not only antiviral drug release can be achieved, but also an enhanced interaction of the nanoparticle with the biological tissue, such as mucous tissue (in the case of mucosal delivery). In general, is known that bacterial activity is exercised by small nanoparticles. However, antiviral activity has been demonstrated for most greater nanoparticles in quantum dots^[^ ^159^ ^]^ and in small nanoparticles for AuNPs,^[^ ^117^ ^]^ therefore size‐dependent antiviral activity must be evaluated for each particular nanoparticle.

From the revised literature, it can be observed the emerging number of recent studies based on the potential of nanoparticles for viral pathologies such as HIV, HVS ‐1, HSV‐2, HBV‐A, HBV‐B, or influenza. However, studies involving nanoparticles and vector‐borne viral diseases are scarce, even though they are a serious problem worldwide. According to reviewed literature, different nanoparticles doted of different elements such as antibodies, drugs, siRNA, NO donors, among others, have demonstrated a higher protection against viruses, acting as antiviral agent or in the promotion of the immune system defense. Still, additional clinical trials must be conducted.

In agriculture, the use the nanoparticles have gained considerable attention in the last years. Nanoparticles of different composition improved plant physiology and growth, enhanced growth parameters under abiotic and biotic stress, including viral infections. The intrinsic properties of nanoparticles have allowed an efficient nanoparticle penetration and translocation, and thus enhancing their antiviral effects in plants, in a similar manner to that in mammals. It should be noted that the antivirus effects can be assigned to the nanoparticle itself (such as in the case of AgNPs) or as a nanocarrier for antiviral agents (such as in the case of polymeric nanoparticles). Although several progresses have been reported in biomedical field, the use of nanoparticles for antiviral purposes in plants is still in its infancy, and more studies are required. Moreover, from the highlighted literature, it is clear that standardized methods are necessary for the evaluation of antiviral activity in plants.

More studies involving unexplored biocompatible and biodegradable nanoparticles with potential against viral infections are required, such as chitosan nanoparticles or PLGA‐NPs. Antimicrobial properties of copper, silver, cerium, or zinc oxides nanoparticles have been demonstrated. However, few studies have been carried out to evaluate their antiviral activity. Moreover, specific parameters (size, concentration, drug concentration, among others) associated with each nanoparticle should be evaluated considering the wide virus diversity as well as the crops. Finally, more studies involving NO‐releasing nanoparticles in plant defense against virus are welcome, considering the key roles of NO in plant, as remarkedly demonstrated in biomedical applications.

Currently, large quantities of nanoparticles are being produced worldwide. From medical research studies, it is possible to affirm that many types of nanoparticles are being synthesized every year, and that their safety and biocompatibility have been actively improving due to the rapid advance of nanobiotechnology. Undoubtedly, many gaps are still unsolved yet, but it is clear that nanobiotechnology in a short time might offer us many nano‐tool alternatives to fight and win the battle against viral diseases in both, human and crop health.

## Conflict of Interest

The authors declare no conflict of interest.
